# Supplementary material for: The Nextflow nf-core/metatdenovo pipeline for reproducible annotation of metatranscriptomes, and more
Source: PeerJ. 2025 Dec 5;13:e20328. doi: 10.7717/peerj.20328 (PMC12684408; doi:10.7717/peerj.20328)
Supplement: Supplemental Information 1 — From left to right the columns are: tool, name of the tool; Version, update versions at the moment of the usage; Citation: references for the tool; Dataset: which dataset used the tool. [file peerj-13-20328-s001.docx]

# Supplementary Table 1. Pipeline Tools, version, citation and dataset in which the tools were used

| **Tool** | **Version** | **Citation** | **Dataset** |
| --- | --- | --- | --- |
| Bbmap /  bbnorm | 39.01 | Bushnell B. BBMap: A Fast, Accurate, Splice-Aware Aligner.  Lawrence Berkeley National Lab. (2014) | MST-1  Kimchi  Eukaryotic |
| Cutadapt | 3.4 | Martin M. Cutadapt removes adapter sequences.  EMBnet.journal (2011) | MST-1  Kimchi  Eukaryotic |
| DIAMOND | 2.1.10 | Buchfink B, Reuter K, Drost HG, "Sensitive protein alignments at tree-of-life scale using DIAMOND", Nature Methods 18, 366–368 (2021). | MST-1 |
| eggnog | 2.1.9 | Cantalapiedra CP, et al. eggNOG-mapper v2:  Functional Annotation for Metagenomics. Mol Biol Evol (2021) | MST-1  Kimchi  Eukaryotic |
| eukulele | 2.0.7 | Krinos A., et al., EUKulele:  Taxonomic annotation of the unsung eukaryotic microbes. (2020, JOSS) | MST-1  Kimchi  Eukaryotic |
| fastqc | 0.12.1 | Andrews S. FastQC: A Quality Control Tool for  High Throughput Sequence Data. (2010) | MST-1  Kimchi  Eukaryotic |
| megahit_interleaved | 1.2.9 | Li D, Liu CM, Luo R, Sadakane K, Lam TW. MEGAHIT:  An ultra-fast single-node solution. Bioinformatics (2015) | MST-1  Kimchi  Eukaryotic |
| Nextflow | 23.10.0 | Di Tommaso P et al. Nextflow enables reproducible  computational workflows. Nat Biotechnol (2017) | MST-1  Kimchi  Eukaryotic |
| prokka | 1.14.6 | Seemann T. Prokka: Rapid prokaryotic genome annotation.  Bioinformatics (2014) | MST-1  Kimchi |
| R | 4.2.3 | R Core Team. R: A language and environment for  statistical computing. R Foundation (2023) | MST-1  Kimchi  Eukaryotic |
| SAMtools | 1.16.1 | Li H. et al. The Sequence Alignment/Map format and SAMtools.  Bioinformatics (2009) | MST-1  Kimchi  Eukaryotic |
| SPAdes | 4.1.0 | E. Bushmanova, et al.,  “rnaSPAdes: a de novo transcriptome assembler and its application to RNA-Seq data”,  GigaScience, 8, 2019, 1–13, doi: 10.1093/gigascience/giz100. | MST-1 |
| Seqtk | 1.4 | Li H. https://github.com/lh3/seqtk | MST-1  Kimchi  Eukaryotic |
| Subread/featureCounts | 2.0.1 | Liao Y., et al., featureCounts:  An Efficient General Purpose Program. Bioinformatics (2014) | MST-1  Kimchi  Eukaryotic |
| TransDecoder | 5.7.1 | Haas B., “TransDecoder.” Jan. 26, 2015. [Online].  Available: https://github.com/TransDecoder/TransDecoder | Eukaryotic |
| TransRate | 1.0.3 | Smith-Unna R et al., TransRate: reference-free quality assessment  of transcriptome assemblies. Genome Research (2016) | MST-1  Kimchi  Eukaryotic |
| Trim Galore | 0.6.7 | Krueger F. Trim Galore: A wrapper around  Cutadapt and FastQC. (2015) | MST-1  Kimchi  Eukaryotic |

# Supplementary Table 2. Pipeline Runs Parameter Settings

| **Parameter** | **Eukaryotic dataset** | **Kimchi dataset** | **MST-1 dataset** |
| --- | --- | --- | --- |
| assembler | - | megahit | Megahit/Spades |
| bbnorm | - | - | True/False |
| diamond_dbs | - | - | ../diamond_dbs.csv |
| eggnog_dbpath | - | ./eggnog-mapper/ | ./eggnog/ |
| eukulele_db | marmmetsp | gtdb | gtdb/phylodb |
| eukulele_dbpath | - | ./eukulele/ | ./eukulele/ |
| input | narragansett_fixed.csv | samplesheet.csv | samples.csv |
| kofam_dir | - | - | ./kofam |
| orf_caller | transdecoder | prokka | prokka |
| outdir | test_metatdenovo | - | megahit.prodigal.with_bbnorm |
| se_reads | - | True | - |
| sequence_filter | - | SILVA_138.1_allrRNAref_NR99_tax.fasta | SILVA_138.1_LSU-SSU_Ref_tax_silva.fna.gz |
| skip_eggnog | - | False | False |
| skip_eukulele | - | False | False |
| skip_fastqc | True | - | - |
| skip_kofamscan | True | True | - |
| skip_qc | True | - | - |
