# Supplementary material for: The Nextflow nf-core/metatdenovo pipeline for reproducible annotation of metatranscriptomes, and more
Source: PeerJ. 2025 Dec 5;13:e20328. doi: 10.7717/peerj.20328 (PMC12684408; doi:10.7717/peerj.20328)
Supplement: Supplemental Information 2 — Parameter settings applied to the Eukaryotic, kimchi and MST-1 dataset. [file peerj-13-20328-s002.docx]

| **Parameter** | **Eukaryotic dataset** | **Kimchi dataset** | **MST-1 dataset** |
| --- | --- | --- | --- |
| assembler | - | megahit | Megahit/Spades |
| bbnorm | - | - | True/False |
| diamond_dbs | - | - | ../diamond_dbs.csv |
| eggnog_dbpath | - | ./eggnog-mapper/ | ./eggnog/ |
| eukulele_db | marmmetsp | gtdb | gtdb/phylodb |
| eukulele_dbpath | - | ./eukulele/ | ./eukulele/ |
| input | narragansett_fixed.csv | samplesheet.csv | samples.csv |
| kofam_dir | - | - | ./kofam |
| orf_caller | transdecoder | prokka | prokka |
| outdir | test_metatdenovo | - | megahit.prodigal.with_bbnorm |
| se_reads | - | True | - |
| sequence_filter | - | SILVA_138.1_allrRNAref_NR99_tax.fasta | SILVA_138.1_LSU-SSU_Ref_tax_silva.fna.gz |
| skip_eggnog | - | False | False |
| skip_eukulele | - | False | False |
| skip_fastqc | True | - | - |
| skip_kofamscan | True | True | - |
| skip_qc | True | - | - |
