# Supplementary material for: The Nextflow nf-core/metatdenovo pipeline for reproducible annotation of metatranscriptomes, and more
Source: PeerJ. 2025 Dec 5;13:e20328. doi: 10.7717/peerj.20328 (PMC12684408; doi:10.7717/peerj.20328)
Supplement: Supplemental Information 3 — Nf-core/metatdenovo removed a high number of contaminants but in proportion it mapped a higher number of reads to the assembly. This proportion doesn’t take into account the taxonomy assignment. For that comparison, see Fig. 7 and the related text. [file peerj-13-20328-s003.docx]

| Database | Sample | Total reads | N. trimmed | N. non contaminated | N. mapped | Percentage |
| --- | --- | --- | --- | --- | --- | --- |
| metatdenovo | J7 | 58133534 | 64716938 | 13904698 | 7782971 | 56,0% |
| metatdenovo | J13 | 70957538 | 76361864 | 14985451 | 10526066 | 70,2% |
| metatdenovo | J18 | 66160090 | 72111904 | 10982168 | 9613398 | 87,5% |
| metatdenovo | J25 | 61382647 | 66736824 | 4065160 | 3000162 | 73,8% |
| metatdenovo | J29 | 63560382 | 69221322 | 6025743 | 4875289 | 81,0% |
| Jung et al | J7 | 58133534 | 30535784 | 20198679 | 425552 | 2,1% |
| Jung et al | J13 | 70957538 | 35269344 | 16650892 | 5332645 | 32,0% |
| Jung et al | J18 | 66160090 | 33894898 | 10820351 | 8442826 | 78,0% |
| Jung et al | J25 | 61382647 | 31437973 | 4250132 | 1768222 | 41,6% |
| Jung et al | J29 | 63560382 | 32562239 | 5793508 | 3869480 | 66,8% |
