# Supplementary material for: The Nextflow nf-core/metatdenovo pipeline for reproducible annotation of metatranscriptomes, and more
Source: PeerJ. 2025 Dec 5;13:e20328. doi: 10.7717/peerj.20328 (PMC12684408; doi:10.7717/peerj.20328)
Supplement: Supplemental Information 4 — Eukrhythmic (columns 2–4) and nf-core/metatdenovo (columns 5–7) for reads mapped back to the assembly. While eukrhythmic uses the Salmon tool against the nucleotide assembly, nf-core/metatdenovo uses the Subread program on predicted open reading frames. Despite this discrepancy, mapping estimates returned by each pipeline were similar as assessed by total number of mapped reads (columns 2 and 5). [file peerj-13-20328-s004.docx]

| Sample | Mapped Reads - eukrhythmic / Salmon | Total Processed Reads - eukrhythmic / Salmon | Percent Mapped - eukrhythmic / Salmon | Mapped Reads - metatdenovo / Subread | Total Processed Reads - metatdenovo / Subread | Percent Mapped - metatdenovo / Subread | Mapped Reads - Alexander et al. 2015 | Total Processed Reads in Mapping - Alexander et al. 2015 | Percent Mapped - Alexander et al. 2015 approach |
| --- | --- | --- | --- | --- | --- | --- | --- | --- | --- |
| S1 | 69678135 | 87912232 | 79.3% | 56601980 | 89455034 | 63.3% | 88941304 | 113595253 | 78.3% |
| S2 | 50058551 | 64550578 | 77.5% | 45870361 | 64888267 | 70.7% | 67317135 | 89670904 | 75.1% |
| S3 | 82211620 | 102399675 | 80.3% | 75906614 | 103250243 | 73.5% | 84690300 | 124842926 | 67.8% |
| S4 | 38096678 | 45297747 | 84.1% | 33248629 | 45370867 | 73.3% | 37558437 | 59249919 | 63.4% |
| S5 | 47708367 | 54877273 | 86.9% | 43716377 | 55061692 | 79.4% | 45575176 | 72938982 | 62.5% |
